# Distribution of Elements in Durum Wheat Seed and Milling Products: Discrimination between Cultivation Methods through Multivariate Data Analysis

**DOI:** 10.3390/foods13121924

**Published:** 2024-06-18

**Authors:** Martina Fattobene, Fuyong Liu, Paolo Conti, Silvia Zamponi, Catia Governatori, Sandro Nardi, Raffaele Emanuele Russo, Mario Berrettoni

**Affiliations:** 1 Chemistry Division, “Chemistry Interdisciplinary Project” Building, School of Science and Technology, University of Camerino, 62032 Camerino, Italy; martina.fattobene@unicam.it (M.F.); fuyong.liu@unicam.it (F.L.); silvia.zamponi@unicam.it (S.Z.); raffaele.russo@unicam.it (R.E.R.); mario.berrettoni@unicam.it (M.B.); 2 Agenzia per l’Innovazione nel Settore Agroalimentare e della Pesca “Marche Agricoltura Pesca” (AMAP), 60035 Jesi, Italy; governatori_catia@amap.marche.it (C.G.); nardi_sandro@amap.marche.it (S.N.)

**Keywords:** cereals, wheat, ICP-OES, spectroscopy, chemometrics, classification

## Abstract

Many staple foods originate from durum wheat and its milling products; because of this, it is very important to know their characteristics. This study investigates elemental contents in these products and if differences exist because of organic farming. The concentrations of 28 elements in the whole seed and in milling products, that is, bran, semolina and flour, of durum wheat, were determined through ICP-OES. The wheats were grown under conventional or organic agronomic practices to verify the possibility of discriminating, using the elemental content, between products coming from one or the other practice. The elements were more abundant in the outer layer of the seed, the bran, but most of them were also present in the others. Traces of Sb were present only in 3% of the samples, while traces of Tl were detected in approximately half of the seed and bran samples but not in other samples. The absence of an element was more characteristic of specific products, e.g., most semolina and flour lacked Co, while other elements showed small differences between products from organic and conventional cultivation or between different milling products, which was the case, for example, for traces of Ag, B, and V. The concentrations of these elements were coupled with multivariate discriminant analysis, specifically PLS-DA, to identify the cultivation provenance of the milled products. A few elements, although different for each product, are sufficient to attain precision and accuracy of classification close to 1; small differences exist for different products. The worst is flour, where the predicted precision and accuracy are 0.92, although using only three elements: B, K, and Se. Semolina attains perfect prediction when also adding to the three previous elements, Ag, Cd, and Cu. Further elements are necessary for bran, while Fe and Mg replace K and Ag to classify seeds. In conclusion, five elements, B, Cd, Cu, K, and Se, are the most important in distinguishing between organic and conventional agriculture; these elements also permit some differentiation among products. The method could help in fraud prevention.

## 1. Introduction

Cereals are among the most important staple food crops; they are a source of calories, proteins and elements for inhabitants worldwide. Among the cereals, durum wheat (Triticum durum) is the basis of many widely used foods, like pasta [1]. The production of durum wheat, the tenth most important crop worldwide, has an important impact on the economy and the environment; it was assessed [2] that these impacts could be improved by organic cultivation practices. The cultivation method affects the final products, but high-quality pasta has been obtained from organic wheat in Southern Italy using selected varieties of wheat [3]. Foods derived from wheat contribute to meeting the body’s need for essential elements [4]; however, when polluted [5,6], they can significantly contribute to overexposure to some elements. Wheat plants exploit elements [7] present in the soil for their biological needs, but the concentration of these elements and their solubility change in different soils, making their uptake by the plants more or less likely [8]. The available content of the elements is related to the contents of clay [9], as shown in Saskatchewan agricultural soils. Another important source of some elements is atmospheric [10] and soil pollution [11]; furthermore, in agricultural practices, there is extensive use of substances containing elements potentially toxic for humans. Wheat plants will therefore accumulate elements influenced by their species and based on different exposures to these aforementioned sources. It is important to understand, since cereal seeds are used for food purposes, how many and which elements are accumulated in the seeds.

The elemental distribution in the kernel is important because from the outer to the inner portions of the seed, during the grinding process, we can obtain bran, semolina and flour, which are used for different kinds of food products. Knowledge of these portions’ elemental affinity can help to produce food with special characteristics or reduce the impact of environmental pollution on the food end-products [12].

While the partitioning of nutrients and minerals among milling products has been investigated [1], to our knowledge, few papers compare the effects of cultivation method on cereal products. These studies are mainly focused on agronomic or environmental comparisons rather than on nutrients in the grains or milled products. The effect of farming methods on nutrient content is still not evident [13]. Marginal differences among nutrients were documented by studying wheat grains from organic and conventional farming methods [14]. A recent comparison of the uptake of Zn and Cd in cases of organic or conventional cultivation methods [15] in grain found no significant differences. However, a multielement investigation focused on the seeds of some crops showed the possibility of some discrimination between crop seeds and their farming method [16].

The accumulation of elements in the seed [1] also depends on the plant genotype, the environment, and the yearly rain amount.

The correlation between the genotypes of common wheat (*Triticum aestivum*) cultivated in China and the mineral element concentrations in their grain was investigated [17] with the goal of selecting genotypes with higher contents of Fe and Zn. Brizio et al. studied the correlation of metals with cereal species in Italy [18], paying attention to micronutrients and toxic elements. A study on French soft wheat showed a connection between topsoil characteristics and the content of metals in common wheat seeds [8]. A comparison of measurements of elements in several varieties of bread and durum wheats grown in Turkey [19] showed high variability in the concentrations. Other important nutrients, like phenolic compounds, are affected by the variety of durum wheat, but their concentration changes within the seed [20].

The geographical traceability of durum wheat has been studied, with elemental analysis being combined with Sr isotopic ratio in a study comparing Italy to the rest of the world [21], and Tyrol cereals [22] being characterized according to ^87^Sr/^86^Sr ratio. 

Multivariate analysis has been applied to several food materials in order to facilitate their authentication and fraud prevention [23]. Some studies involve cereals for which infrared spectroscopy [24] coupled with calibration methods permits the measurement of chemical composition (e.g., protein, moisture, oil), while the same spectroscopic techniques combined with pattern recognition and/or discriminant techniques were used for the authentication and traceability of cereals. Some works consider volatile substances, chromatographically determined, as the basis for multivariate methods for classification according to wheat cultivation area and species [25] or to authenticate Italian pasta [26] or even to correlate characteristics to cultivation altitude [27]. There are studies in which chemometric methods are coupled with element measurements for the purpose of the authentication of agricultural products [28,29]. 

The present work aims to expand knowledge about the content of the elements present in the various durum wheat products: seed, bran, semolina and flour. This study includes many varieties grown according to organic and conventional protocols to verify if there is a difference, at the ground product level, between these two cultivation protocols. To this end, some chemometric methods will be needed to analyze the set of measurements.

## 2. Materials and Methods

### 2.1. The Samples

The seeds of different varieties of durum wheat were sampled after their harvesting in July 2022; they were stored in a refrigerator until their grinding. The wheat was cultivated in the experimental fields of the Agenzia per l’Innovazione nel Settore Agroalimentare e della Pesca “Marche Agricoltura Pesca” (AMAP) in Jesi, in two fields not far from each other, one devoted to organic [30] cultures and the other to conventional farming. These are clay soils; each field is divided into parcels of 7 × 1.4 m, in each of which a different variety of wheat is grown. Every variety is replicated on three parcels. The seed harvest is carried out by keeping the seeds from each plot separate. 

The plots in conventional agriculture were weeded with Syngenta Amadeus Top (50 g/ha), containing the active substances thifensulfuron-methyl and tribenuron, and Syngenta Traxos Pronto 60 (1 l/ha), containing active ingredients pinoxaden and clodinafop. Fertilization was carried out with ammonium nitrate at a rate of 148 kg/ha on 17th February 2022, and 304 kg/ha on 14th April 2022. 

To respect the strict rules about organic production (Regulation (EU) 2018/848 of the European Parliament and of the Council of 30 May 2018) and the admitted substances (Commission Implementing Regulation (EU) 2021/1165 of 15 July 2021, amended with Commission Implementing Regulation (EU) 2023/121 of 17 January 2023), no weeding was performed in the organic crops. Fertilizations were carried out with Italpollina Hello Nature products: 200 kg/ha of Guanito in pre-sowing on 29 October 2021 and 400 kg/ha of DIX N10 on 17 February 2022, buried with a harrow. 

The seeds were milled to obtain four products from each variety: whole seed, bran, semolina, and flour, as detailed below. Some additional seed samples were not milled. Table 1 provides details of the samples.

In the following coding scheme, every product sample is coded with the following syntax: CC_ntt, where CC is the code indicated in the second column of Table 1, n indicates the agricultural method, with 1 representing conventional and 2 representing organic. The two characters tt are absent in the seed codes, while they are Cr for bran, Se for semolina, and Fa for flour.

### 2.2. Milling

The durum wheat seed samples were conditioned to reach 17% humidity by adding water in two stages: 16 h and 3 h before grinding. The seeds were then milled using a CD2 Chopin Technologies mill and then passed through the Chopin purifier to obtain three fractions:

Flour: ≤160 μm;
Semolina: 160 < semolina ≤ 560 μm;
Bran: >560 μm.

The conditioning of the grain is a practice carried out by laboratories and mill facilities to facilitate the separation of the endosperm from the cortical part, aiming to obtain a greater yield of flour/semolina. Since there is no official method, everyone sets the parameter values following their protocol. Here, the internal protocol of the “Sperimentazione e Monitoraggio dell’Innovazione delle colture agrarie” laboratory of AMAP was followed, which is nonetheless similar to the procedure of other authors [20].

### 2.3. Mineralization

The samples of whole seeds, bran, semolina, and flour were dried in an oven at 60 °C for 24 h. The seeds were washed with ultrapure 18.2 MΩ water from a Milli-Q (Millipore, St. Louis, MO, USA) before drying. Approximately 1 g of each dry sample (whole seeds, bran, semolina, flour) was mixed with 8 mL ultrapure HNO_3_ 65% and 2 mL ultrapure HCl 37%, then digested using a microwave-assisted instrument (ultraWAVE, Milestone Srl, Sorisole (BG), Italy) for 40 min. The digests were recovered with ultrapure water and diluted to 50 mL. All the reagents are high-purity Merck products for Inductively Coupled Plasma Spectroscopy (ICP). 

### 2.4. ICP Analysis

The mineralized solutions were analyzed by a ThermoFisher Scientific (Waltham, MA, USA) iCAP PRO X Duo ICP-OES for the elements listed in Table 2. Quantification occurred using a five-point calibration line for each element, ranging from 0.001 to 10 mg/L in decadic increments. Calibrations were obtained using the Multi Element Standards IQC-026 (Ultra Scientific Italia, Bologna, Italy), with the exceptions of P (Sigma Aldrich 207357, Kawasaki City, Japan) and Sn (Merck 43922907, Rahway, NJ, USA). The Limit of Detection (LOD) and Limit of Quantification (LOQ) were automatically computed from the calibration lines by the instrumental software. Samples outside the calibration range were suitably diluted to fall within the calibration range.

Complementary analysis of soil samples was executed as described in Appendix A.

### 2.5. Processing of the Results: Methods and Softwares

The acquired data were analyzed to disclose the difference in the elemental content of the various products and then to check if a difference exists between samples from organic agricultural practices and those from conventional agricultural practices. For this purpose, descriptive statistics for every element were performed using The Unscrambler^®^ X software (version 10.2, CAMO Software, Oslo, Norway). The same software was used to study the relations between the elements and the products, as well as those between elements and farming protocols, using Principal Component Analysis (PCA). Discriminant analysis with the PLS-DA algorithm and variable selection were performed using The Unscrambler. Subsequent refinement was conducted in Matlab^®^ (version R2023a, The MathWorks Inc., Natick, MA, USA) with class_gui [31] toolbox. Microsoft Office^®^ Excel was utilized for data arrangement and elementary statistics. 

The data were arranged, for the multivariate analysis, in a data matrix where each column contains the concentration values of a different element (variable), and each row represents the concentrations of the elements in a sample (object). The data were analyzed first using Principal Component Analysis (PCA) to screen the importance of the elements, followed by supervised PLS-DA to check the discrimination ability with respect to the cultivation method. PCA [32] is part of the tools used for a better understanding of the analytical results regarding complex matrices such as food. It permits condensation of the main information into a reduced variable space. However, when discrimination of groups is required, Partial Least Squares Discriminant Analysis (PLS-DA) [33] is often chosen as a supervised classification method. PLS-DA algorithm is a Partial Least Squares regression method where an independent block of variables is related to a dependent block of variables. In the case of discriminant techniques, the dependent variables are dummy variables, each associated with a class, whose values indicate the belonging or no–belonging of the sample to the class. The prediction ability of the method is generally estimated by cross-validation [34,35], which permits for the simulation of an unknown data set (evaluation set).

PCA orthogonally rotates the variables to obtain the projection (scores) of the samples in a reduced space, that of principal components (PCs), where relations are more easily visible. The loadings of variables indicate the importance of the related variable with respect to the component. The concentrations of the elements were the independent block variables of PLS-DA, while two dummy variables, one for the organic category and the other for the conventional ones, formed the dependent block. These variables are coded as 0 and 1, respectively, to indicate whether the sample belongs to the associated class [33]. The trials initially consider all the product samples as a whole and then each product separately. The most important elements for each PLS-DA analysis were selected. The quality of the PLS-DA models was assessed using the percentage of variance explained (R^2^) by the PLS model and the analogous value in prediction (Q^2^), evaluated with 5 group cross-validation using The Unscrambler X. The accuracy (percentage of correct classifications over the total number of samples) and precision (percentage of correctly assigned samples among those predicted in the class) values were evaluated using the classification toolbox for Matlab [31].

The measured values were autoscaled before the multivariate analysis. In many of these trials, the values were log_10_ transformed before autoscaling due to the high concentration differences among the elements. Comparing data treatments, including simple autoscaling of the variables or logarithmic transformation followed by autoscaling, showed similar or worse results for the former.

Variable selections to optimize PLS-DA results were performed with The Unscrambler X, which applies Martens’s uncertainty test [36].

The correlation figure in the Supplementary Materials was obtained by PAST version 4.14 software (P. D. R. Øyvind Hammer David A. T. Harper, “Past: Paleontological Statistics Software Package for Education and Data Analysis”, Palaeontologia Electronica, vol. 4, no. 1, p. 9, 2001). The data treatment involved 13 samples in both categories (organic and conventional) for bran, semolina, and flour. When seeds were analyzed, there were 19 organic samples and 17 conventional samples.

## 3. Results and Discussion

The 28 elements were measured in four wheat products: whole seed, bran, semolina, and flour. Table S1 shows the LOD, LOQ, mean, median, standard deviation (SD), and range for each element across all sample types (seed, bran, semolina, and flour considered as a unique set) grouped by cultivation protocol. Similar analyses were performed on each product, and the results are summarized in Table 2 for whole seeds, Table 3 for bran, Table 4 for semolina, and Table 5 for flour. Some elements are present in trace amounts and may occasionally be absent in one or more of the products. The Sb has values ≤LOD in 93% of the samples, with 97% of them ≤LOQ. The Tl is not present in semolina and flour (~93% of samples <LOD). Tl has values >LOD in 50% of seed samples and 58% of bran samples, but most of the positive samples have concentrations close to LOD. Co was under the detection limit (43% < LOD, 58% < LOQ) of samples, especially due to its absence in most semolina (96% < LOD) and flour (81% < LOD) samples Most of organic samples contain slightly less Co than the conventional ones. Beryllium (Be) was not detected in 67% of the whole seed samples, but traces are present in bran, semolina, and flour. Boron (B) is not detectable in nearly half of the organic semolina samples (46% < LOD) and in some organic flour samples (~8% < LOD). However, it is measurable in organic seeds, bran, and in all conventional samples. Ag, when detectable, has values close to its LOQ; it is present in bran, some seed samples (39% of seeds <LOQ), and a few samples of semolina (43% < LOD) and flour (46% < LOD). The measurements of V are <LOD in approximately 15% of the bran and semolina samples. All other elements were detected in all samples.

As shown in Table S1, there are minor differences between values obtained from organic samples and those from conventional cultivation. The values obtained are comparable with those found in the literature. The range of values for Cd, Cu, and Zn are similar to those reported for wheat grown in Marche [37]. The B content is similar to that measured in Austrian wheats [38] but lower than the values measured in wheat grown in Saskatchewan [9]. As content in our samples is slightly higher than the values reported by Cubadda et al. [39]. 

The present study focuses on the potential to identify the cultivation protocol used for the wheats under investigation through simple analysis and multivariate treatment. Several varieties of wheats were cultivated under controlled conditions in a restricted experimental area, meaning that some sources of variability, such as the season effects, humidity, and soil differences, were not considered. Reducing these sources of variance under controlled conditions is important for a new trial like this, where the main focus is on a specific characteristic: the farming method. Consequently, this study is a methodological experiment that requires further investigation to extend its use in real-world conditions. This study develops a potentially useful method for protecting foodstuffs, but it needs further validation with a broader database includes the sources of variability not considered here.

### 3.1. Descriptive Statistics

The effect of the cultivation method was initially evaluated by performing an equal variance two-tailed *t*-test (α = 0.05) for each element. The averages were calculated from the values obtained from the samples grouped according to the farming method: organic or conventional. This test was performed on all products as a whole and individually for each product. The significantly different elements (*p*-value ≤ 0.05) are indicated in the tables with a double asterisk.

Comparing organic products to conventional ones using a *t*-test provides limited information. Most of the elements show no significant difference, though some test positively for certain products, as highlighted in Table 2, Table 3, Table 4 and Table 5, where the main parameters are also reported. Each element such as Ba, Be, Co, Cr, Mn, Ni, and Zn that are significantly different in soils (Table A1 in Appendix A), have no difference in the products. While V which is significant in soil, shows a significant difference only in Semolina. The other elements, including Al, Cd, Cu, and Fe, which are significant in soil, also show significant differences in most of the products. B and Se have significant *t*-tests in all products, while K and Al are significantly different in all milled products.

Figure S1 shows the correlation between elements within the products. Strong correlations are evaluated for Zn, Mg, Mn, P, K, and other elements, some of which linked to one or more products. These correlations suggest a relationship between the elements, which may indicate a common origin, related biochemistry, or other, possibly unknown relations. 

### 3.2. Pattern Recognition

The comparisons of the element contents reported in Table 2, Table 3, Table 4 and Table 5 show some differences between grains grown using conventional and organic cultivation methods. However, these differences in the amount of the elements, also if present, are confounded by high variability in the values. Consequently, univariate analysis of the data does not clearly distinguish between the materials (seed, bran, semolina, and flour) or the farming methods (organic or conventional). A multivariate approach could therefore simplify the interpretation of the results.

Since Sb and Tl are not present in most of the samples, they were excluded from the data treatment.

**Figure 1:**
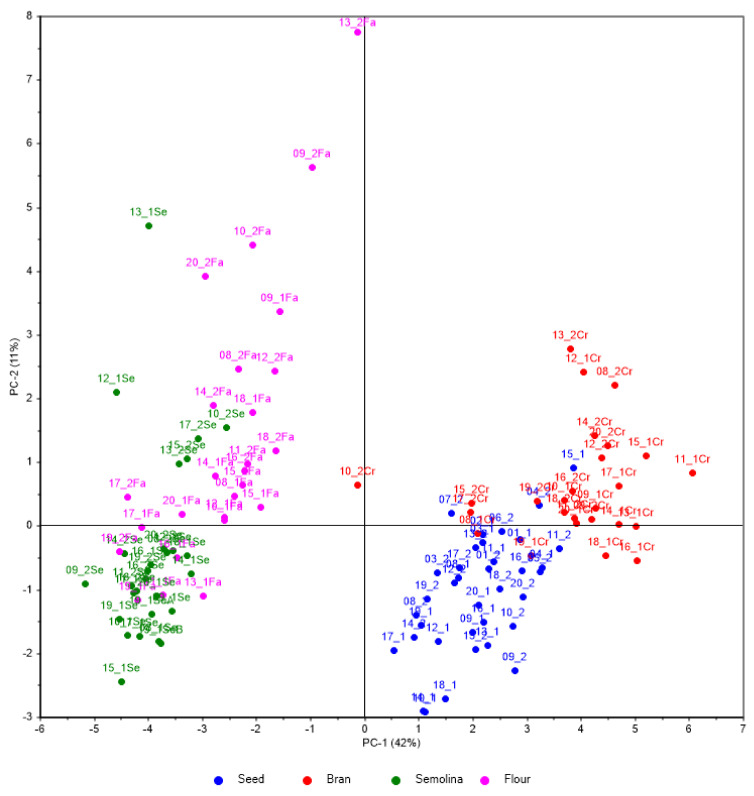
Scores projection of the first two components computed on log_10_ transformed and autoscaled data. All material samples were included.

The PCA analysis of the obtained values (see Figure 1), despite their similarity, highlights that the first component (42% of explained variance) separates two groups: seed and bran have high values on PC1, while semolina and flour are positioned on the opposite side of PC1. With the help of the second PC (11% of explained variance), seed and bran are separated, although semolina and flour show more overlap. These results are anticipated because semolina and flour are both derived from the kernel of the seed and consist mainly of starch. Bran, being the outer layer of the seed, is expected to have a different elemental content due to its distinct structure [1]. The PCA confirms that the elemental content in bran is largely similar to that of the whole seeds, consistent with documented accumulation of most elements in the bran [1].

**Figure 2:**
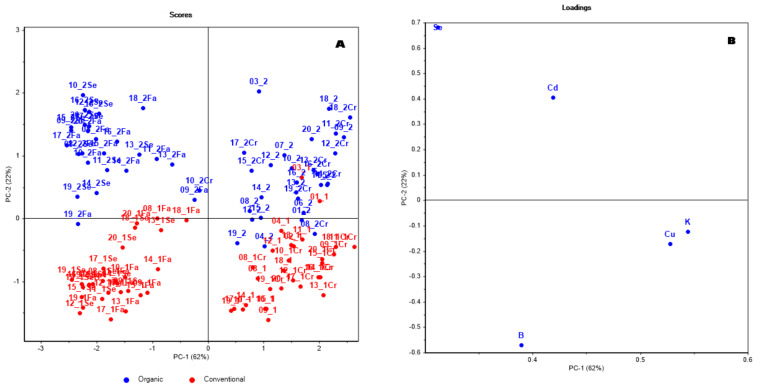
Projection of the first two components computed on autoscaled data after variable selection, colors highlight the cultivation methods. Box (**A**) scores and (**B**) loadings.

Figure 2 highlights the cultivation protocol on the PCA projection of autoscaled data, where PC1 and PC2 account for 62% and 22% of the variance, respectively. The figure shows that a discernible difference between samples from organic and conventional farming methods. This distinction seems more pronounced in the milled portions (bran, semolina, flour) than in the whole seed. It is noteworthy that this result follows the selection of a reduced number of elements: B, Cu, K, Cd, and Se. The separation of the organic samples from conventional ones primarily correlates with Se, B, and Cd, whereas differences between the product types are additionally affected by Cu and K.

### 3.3. Classification

PCA suggests the possibility of discriminating between organic and conventionally cultivated samples. Therefore, supervised PLS-DA discriminant analysis was used to optimize and quantify this result. For each analysis, the variable selection was optimized. Table 6 shows the percentage of variance explained (R^2^) by the PLS-DA model and the analogous prediction value (Q^2^), evaluated with five groups of cross-validation. Table 6 also reports the selected elements in each dataset necessary to obtain the optimized classification, along with the number of latent variables required to achieve these results. Parameters are provided to estimate the ability to predict the training objects, with corresponding “prediction” value to assess the predictive ability of unknown samples simulated by cross-validation (evaluation set). For effective classification performance, it is desirable to have similar values of the parameters estimated from both the training set and the evaluation one. The PLS-DA analysis uses the log_10_ transformed and autoscaled values of the elements as predictors. However, very good results are also obtained with simply autoscaled measured values, as shown by the comparisons in Table 6. PLS-DA effectively classifies products according to the cultivation methods. For each product, simple models are generally sufficient; discrimination in any milled product is generally achieved with only one or two latent variables, as reported in the fourth column of Table 6.

The results shown in Table 6 are from the analysis with the optimized number of variables that are indicated in the same table.

Table 6 highlights that the poorest classification is observed for seeds, which appears to yield a less stable model. On the contrary, Bran, Semolina, and Flour show clear differentiation between organic and non-organic samples. Semolina stands out with an R^2^ of 0.91 and Q^2^ of 0.89. Both bran and semolina have 100% accuracy and precision while the Flour shows a prediction accuracy of 96%. Notably, using data without log_10_ transformation yields similar performance but with an increased number of retained elements. Considering all the samples together, the average result is consistently good. One latent variable is enough for most of the classification models, with some requiring two or more. Comparable discrimination results are achieved using alternative discriminant methods. Table 6 also compares the effect of log transform. It is noteworthy that the transform moderately enhances the classification ability, while the important elements remain largely consistent across most comparisons, except for Bran, where the log transformation allows a reduction in the number of required elements. Only a few elements significantly contribute to the discrimination, indicating their sensitivity to farming methods, which varies based of the product. B and Se appear consistently in every model indicating their importance in farming differentiation. K is crucial across all the milled fractions, while Cd consistently included among the selected elements except in the suboptimal Flour model, where B, K, and Se suffice for accurate and precise differentiation Cu is always selected except in Flour with log_10_ transformation.

Some elements: As, Ba, Be, Ca, Mn, Ni, Ti, and Zn never entered the selected variables, which indicates they are unaffected by the cultivation method Moreover, Sb and Tl were not included in the analysis because of their absence in most of the samples, as previously noted. Mg is selected only when analyzing the seeds without log transformation, while V and Al are included in the selected variables only when analyzing flour without log transformation. Si is selected under these conditions as well, and it is selected when analyzing all the samples without transformation. Ag is selected only when analyzing semolina without transformation. Co, Na, Pb, and Sn are associated to bran, with the addition of Mo and P if only autoscaling is applied.

Few elements, B, Cd, Cu, K, Se are the most meaningful as they permit to differentiation of the products based on cultivation protocols. PCA with these five elements also reveals distinct grouping based on the products. Despite metals being more abundant in the outer layer (pericarp and aleurone) of the seed [41], some are absorbed differently into the kernel, enabling discrimination even between semolina and flour on cultivation protocols. The large differences in elemental content due to phenotypes do not affect the discrimination ability concerning the kind of cultivation protocol. 

The high concentration of some elements in soil, like Al, Cr, Fe, and Cu, appears not very effective on the final products. In fact, Al and Cr are included only in the classification model of flour, but even without them, the classes of flour can be distinguished well enough. Fe and Cu are included in the models of seed and bran, with Cu also playing an important role in the classification of semolina. The simplest model obtained is the suboptimal one for flour, which requires only three elements, B, K, and Se to discriminate between organic and conventional flour. 

Models with very high accuracy and precision, both 100%, are obtained for every milled product. In the case of flour, simplifying the model with three elements reduces these values to an optimum 92% for both parameters. Bran is the material that needs the largest number of elements to achieve good classification models, probably due to the concentration of many elements in its outer layer, which is the outer layer of the seed. The models for seeds are slightly simpler than those for bran, which may seem contradictory; however, the seeds include all the other milled products, with bran being rich in elements and the others being less so. Therefore, the models for seeds can be seen as an average between the simplest model for flour and the more complex models for bran and semolina. The reduced number of elements that affect the discrimination of the refined products derived from wheat grown under organic or conventional cultivation align with the distribution of the elements in the wheat seed. In fact, most of the elements concentrate in the outer layer, while their concentration decreases in the inner parts from which semolina and flour are derived. 

To the best of our knowledge, only a few studies address differences in element content due to the type of cultivation, generally not detecting significant variations among different elements. Laursen and co-authors investigated the possibility of obtaining an element-based fingerprint to distinguish cereal species, geographical origin, and cultivation methods [16]. Their analysis, mainly based on PCA, showed limited ability to discriminate between wheat seed cultivation methods.

## 4. Conclusions

This study characterizes the elemental content of milled products from various durum wheats grown in Italy by ICP-OES measurements. This instrumentation is widely available in the analytical laboratories, allowing for cost-effective measurements that are useful for advanced data processing. The availability of a multistandard elements makes the technique efficient and rapid for measuring multiple elements simultaneously. PLS-DA is a widely used data processing algorithm that does not require highly specialized infrastructure. 

The measurements were used to discriminate the milled durum wheat products based on the cereal cultivation protocol. A data processing methodology is proposed to obtain discrimination using the elemental quantities present in the products. The results are highly promising, especially for semolina and bran (100% precision and accuracy), but even flour can be classified with optimal precision and very high accuracy. These results were obtained with PLS-DA, which performs slightly better than other discriminant algorithms.

An important finding is that the discriminations are due to a minimum of three elements (B, K, Se), but even the products requiring a few more elements can be classified with fewer elements if a slightly lower classification accuracy is acceptable. In this context, B, Cd, Cu, K, and Se are the most effective elements.

The investigated method, due to its ability to differentiate between organic and conventional products, could be applied to prevent commercial fraud.

## Figures and Tables

**Table 1:** Details of the samples.

Wheat Variety	Code of the Variety	Code of the Organic Samples	Code of the Conventional Samples	Seed	Bran	Semolina	Flour
**Saragolla new**	01	01_2	01_1	Yes	NO	NO	NO
**San Carlo**	02	NO	02_1	Yes	NO	NO	NO
**Fuego grown in a large plot**	03	03_2	03_1	Yes	NO	NO	NO
**EVOLDUR evolutionary population harvest 2021**	04	04_2	NO	Yes	NO	NO	NO
**Evoldur grown in a large plot**	04	NO	04_1	Yes	NO	NO	NO
**Senatore Cappelli**	05	05_2	NO	Yes	NO	NO	NO
**Saragolla old**	06	06_2	NO	Yes	NO	NO	NO
**Fuego grown in the edge near the railway**	07	07_2	NO	Yes	NO	NO	NO
**Antalis**	08	08_2	08_1	Yes	Yes	Yes	Yes
**Bering**	09	09_2	09_1	Yes	Yes	Yes	Yes
**Casteldoux**	10	10_2	10_1	Yes	Yes	Yes	Yes
**Claudio**	11	11_2	11_1	Yes	Yes	Yes	Yes
**Fuego**	12	12_2	12_1	Yes	Yes	Yes	Yes
**Idefix**	13	13_2	13_1	Yes	Yes	Yes	Yes
**Iride**	14	14_2	14_1	Yes	Yes	Yes	Yes
**Marakas**	15	15_2	15_1	Yes	Yes	Yes	Yes
**Marco Aurelio**	16	16_2	16_1	Yes	Yes	Yes	Yes
**Monastir**	17	17_2	17_1	Yes	Yes	Yes	Yes
**Platone**	18	18_2	18_1	Yes	Yes	Yes	Yes
**RGT Natur**	19	19_2	19_1	Yes	Yes	Yes	Yes
**Tito Flavio**	20	20_2	20_1	Yes	Yes	Yes	Yes

**Table 2:** Comparison of seeds (** *p* ≤ 0.05 and * *p* ≤ 0.01) indicates the elements whose means give a significant two-tailed *t*-test comparison between the organic (19 samples) product and the conventional (17 samples) one. Values of 0.000 in means and ranges indicate measurable elements, >LOQ, but with very low amounts. LOD and LOQ are used to indicate a value ≤LOQ (presence of trace) or a value ≤LOD (not detectable). The scale of the values is mg/kg dry weight.

		Organic Seeds		Conventional Seeds	
Element	*p*-Value (t)	Mean ± SD	Min–Max	Mean ± SD	Min–Max
**Ag**	0.161 (1.433)	0.003 ± 0.002	LOD–0.006	0.003 ± 0.002	LOD–0.009
**Al**	0.375 (0.900)	4.811 ± 1.522	1.788–6.583	4.216 ± 1.897	1.533–6.741
**As**	0.736 (0.340)	0.073 ± 0.031	0.024–0.156	0.075 ± 0.043	0.020–0.218
B (**)	0.000 (3.876)	2.107 ± 0.405	0.526–2.502	2.686 ± 0.453	1.999–3.185
**Ba**	0.464 (0.741)	0.842 ± 0.357	0.358–1.631	0.756 ± 0.282	0.394–1.359
**Be**	0.160 (1.436)	LOD ± 0.001	LOD–0.001	0.000 ± 0.000	LOD–0.001
**Ca**	0.316 (1.017)	482.951 ± 23.743	432.194–519.332	472.957 ± 35.970	392.386–531.502
Cd (*)	0.021 (2.420)	0.029 ± 0.010	0.016–0.045	0.022 ± 0.007	0.013–0.036
**Co**	0.936 (0.081)	0.005 ± 0.003	LOD–0.011	0.005 ± 0.003	LOQ–0.009
**Cr**	0.433 (0.793)	0.084 ± 0.010	0.072–0.115	0.109 ± 0.131	0.064–0.615
Cu (*)	0.018 (2.495)	4.091 ± 0.534	3.433–5.226	4.610 ± 0.537	3.851–5.814
Fe (**)	0.002 (3.277)	28.111 ± 2.702	23.929–34.350	25.455 ± 2.115	21.324–29.221
**K**	0.289 (1.077)	3203.997 ± 176.816	2933.319–3697.111	3287.373 ± 157.776	3046.468–3587.377
Mg (*)	0.025 (2.338)	526.603 ± 18.206	481.189–558.077	513.229 ± 18.949	484.819–553.452
**Mn**	0.298 (1.056)	35.561 ± 4.322	28.101–42.808	34.665 ± 4.715	28.301–41.289
**Mo**	0.385 (0.881)	0.929 ± 0.215	0.566–1.327	1.001 ± 0.296	0.659–1.644
**Na**	0.518 (0.654)	27.683 ± 7.748	18.349–48.180	25.580 ± 4.584	20.586–37.991
**Ni**	0.649 (0.459)	0.177 ± 0.059	0.111–0.327	0.198 ± 0.110	0.105–0.580
**P**	0.542 (0.615)	3373.664 ± 268.498	2984.810–3903.900	3341.108 ± 245.271	3001.920–3889.520
**Pb**	0.522 (0.646)	0.057 ± 0.018	0.036–0.092	0.054 ± 0.025	0.026–0.128
**Sb**	0.606 (0.521)	LOQ ± 0.005	LOD–0.019	LOD ± 0.005	LOD–0.020
Se (**)	0.000 (5.357)	0.238 ± 0.036	0.151–0.290	0.178 ± 0.029	0.131–0.222
**Si**	0.927 (0.093)	22.694 ± 5.045	13.869–32.932	22.010 ± 8.297	13.517–48.981
**Sn**	0.821 (0.228)	0.005 ± 0.002	0.003–0.009	0.005 ± 0.003	0.002–0.011
**Ti**	0.830 (0.216)	0.021 ± 0.013	0.007–0.064	0.020 ± 0.011	0.007–0.039
**Tl**	0.196 (1.320)	0.004 ± 0.007	LOD–0.022	0.010 ± 0.013	LOD–0.043
**V**	0.439 (0.784)	0.009 ± 0.002	0.006–0.011	0.010 ± 0.002	0.006–0.015
**Zn**	0.349 (0.950)	30.130 ± 5.757	22.541–45.556	28.863 ± 3.997	24.495–40.231

**Table 3:** Comparison of brans. (** *p* ≤ 0.05 and * *p* ≤ 0.01) indicates the elements whose means show a significant difference in a two-tailed *t*-test comparison between the organic and conventional products. Both groups in the *t*-test consisted of 13 samples. LOD and LOQ indicate values ≤LOQ (presence of trace) or values ≤LOD (not detectable). The values are reported in mg/kg dry weight.

		Organic Brans		Conventional Brans	
Element	*p*-Value (t)	Mean ± SD	Min–Max	Mean ± SD	Min–Max
**Ag**	0.132 (1.559)	0.005 ± 0.002	LOQ–0.007	0.007 ± 0.003	LOQ–0.013
Al (*)	0.040 (2.167)	5.916 ± 2.512	3.784–12.263	4.270 ± 0.797	2.579–5.757
**As**	0.278 (1.111)	0.070 ± 0.025	0.021–0.118	0.088 ± 0.052	0.045–0.236
B (**)	0.001 (3.781)	1.504 ± 0.340	0.578–1.890	1.919 ± 0.139	1.561–2.092
**Ba**	0.588 (0.549)	1.093 ± 0.379	0.603–1.953	1.181 ± 0.440	0.587–2.057
**Be**	0.337 (0.980)	0.001 ± 0.000	0.001–0.001	0.001 ± 0.001	0.001–0.003
Ca (*)	0.045 (2.118)	465.731 ± 23.447	412.787–490.929	486.383 ± 26.193	447.286–525.940
**Cd**	0.099 (1.714)	0.031 ± 0.012	0.017–0.053	0.024 ± 0.007	0.017–0.035
**Co**	0.067 (1.915)	0.004 ± 0.002	LOD–0.008	0.006 ± 0.003	LOQ–0.012
**Cr**	0.298 (1.063)	0.104 ± 0.020	0.072–0.140	0.112 ± 0.015	0.087–0.137
Cu (**)	0.000 (4.295)	4.925 ± 0.712	3.456–5.848	6.168 ± 0.763	5.078–7.457
**Fe**	0.996 (0.005)	36.673 ± 6.217	25.335–48.132	36.684 ± 3.136	30.115–41.784
K (**)	0.006 (3.005)	3702.171 ± 516.030	2561.379–4242.568	4206.877 ± 283.921	3511.909–4540.521
**Mg**	0.137 (1.538)	558.948 ± 36.235	462.179–603.480	576.091 ± 15.827	547.554–603.324
**Mn**	0.063 (1.953)	46.950 ± 9.574	24.625–61.671	54.534 ± 10.216	35.295–73.585
Mo (**)	0.001 (3.794)	0.980 ± 0.166	0.733–1.258	1.264 ± 0.213	0.973–1.598
Na (*)	0.026 (2.378)	35.491 ± 9.294	24.952–61.069	49.927 ± 19.203	34.075–97.760
**Ni**	0.574 (0.570)	0.223 ± 0.090	0.086–0.419	0.241 ± 0.063	0.147–0.343
P (**)	0.010 (2.817)	3974.312 ± 596.423	2544.200–4748.610	4520.654 ± 365.057	3664.670–4985.480
Pb (*)	0.012 (2.707)	0.100 ± 0.027	0.055–0.135	0.077 ± 0.016	0.055–0.106
**Sb**	0.385 (0.885)	LOD ± 0.001	LOD–LOD	LOD ± 0.001	LOD–LOD
Se (**)	0.000 (9.601)	0.242 ± 0.024	0.188–0.275	0.166 ± 0.015	0.141–0.195
**Si**	0.790 (0.269)	33.757 ± 12.361	14.746–56.431	35.167 ± 14.267	19.242–69.089
Sn (*)	0.027 (2.350)	0.007 ± 0.003	0.004–0.016	0.005 ± 0.001	0.003–0.009
**Ti**	0.240 (1.204)	0.061 ± 0.040	0.019–0.153	0.046 ± 0.021	0.023–0.099
**Tl**	0.969 (0.039)	0.007 ± 0.010	LOD–0.033	0.007 ± 0.007	LOD–0.021
**V**	0.473 (0.730)	0.009 ± 0.006	LOQ–0.021	0.008 ± 0.006	LOQ–0.018
**Zn**	0.079 (1.835)	37.297 ± 6.372	22.804–45.948	41.169 ± 4.154	33.005–47.971

**Table 4:** Comparison of Semolina. (** *p* ≤ 0.05 and * *p* ≤ 0.01) indicates the elements whose means show a significant difference in a two-tailed *t*-test comparison between the organic and conventional products. Both groups in the *t*-test have 13 samples. LOD and LOQ indicate values ≤LOQ (presence of trace) or values ≤LOD (not detectable). The values are reported in mg/kg dry weight.

		Organic Semolina		Conventional Semolina	
Element	*p*-Value (t)	Mean ± SD	Min–Max	Mean ± SD	Min–Max
**Ag**	0.074 (1.871)	LOQ ± 0.001	LOD–0.005	0.003 ± 0.002	LOD–0.007
Al (*)	0.023 (2.436)	6.147 ± 2.871	3.461–13.392	3.662 ± 1.678	1.447–7.705
**As**	0.459 (0.753)	0.070 ± 0.038	0.027–0.182	0.083 ± 0.081	0.024–0.363
B (**)	0.000 (6.983)	0.288 ± 0.432	LOD–0.951	1.346 ± 0.188	1.109–1.851
**Ba**	0.121 (1.606)	0.482 ± 0.125	0.321–0.767	0.395 ± 0.109	0.279–0.635
**Be**	0.337 (0.980)	0.001 ± 0.000	0.001–0.001	0.001 ± 0.000	0.001–0.002
**Ca**	0.524 (0.647)	437.659 ± 28.502	404.677–486.215	430.917 ± 31.543	390.838–479.139
Cd (*)	0.024 (2.414)	0.017 ± 0.006	0.010–0.031	0.012 ± 0.004	0.008–0.018
**Co**	0.386 (0.882)	LOD ± 0.002	LOD–0.009	LOD ± 0.000	LOD–LOD
**Cr**	0.309 (1.039)	0.057 ± 0.051	0.034–0.226	0.042 ± 0.009	0.024–0.061
Cu (**)	0.008 (2.871)	2.341 ± 0.226	2.034–2.745	2.614 ± 0.281	2.138–3.228
Fe (**)	0.007 (2.950)	13.298 ± 2.761	9.983–19.124	10.612 ± 1.404	7.844–13.894
K (**)	0.004 (3.138)	1657.293 ± 134.168	1377.052–1864.274	1808.033 ± 112.416	1623.165–2009.210
**Mg**	0.289 (1.085)	349.486 ± 24.248	300.566–383.394	341.154 ± 15.061	314.100–365.416
**Mn**	0.216 (1.270)	8.885 ± 0.868	7.173–10.532	9.391 ± 1.373	7.456–11.745
**Mo**	0.349 (0.955)	0.810 ± 0.210	0.539–1.249	0.855 ± 0.131	0.702–1.101
**Na**	0.278 (1.111)	21.091 ± 4.336	16.225–32.199	31.682 ± 23.065	15.214–95.377
**Ni**	0.654 (0.454)	0.074 ± 0.102	0.027–0.411	0.088 ± 0.071	0.041–0.330
**P**	0.238 (1.209)	1506.895 ± 135.215	1286.050–1710.380	1577.838 ± 123.078	1337.920–1710.160
**Pb**	0.556 (0.597)	0.069 ± 0.014	0.054–0.102	0.077 ± 0.054	0.038–0.263
**Sb**	0.935 (0.083)	LOD ± 0.001	LOD–LOD	LOD ± 0.002	LOD–LOQ
Se (**)	0.000 (6.063)	0.191 ± 0.025	0.155–0.240	0.118 ± 0.032	0.077–0.212
Si (**)	0.003 (3.285)	11.120 ± 3.979	6.314–19.054	7.135 ± 1.866	4.997–11.171
**Sn**	0.349 (0.954)	0.007 ± 0.002	0.005–0.010	0.010 ± 0.014	0.004–0.059
**Ti**	0.665 (0.439)	0.033 ± 0.018	0.016–0.077	0.028 ± 0.018	0.008–0.077
**Tl**	0.258 (1.158)	LOD ± 0.000	LOD–LOD	LOQ ± 0.001	LOD–0.003
**V**	0.279 (1.108)	0.008 ± 0.006	LOQ–0.020	0.046 ± 0.132	LOQ–0.520
**Zn**	0.200 (1.319)	10.907 ± 0.955	8.952–12.198	11.478 ± 1.072	9.035–12.972

**Table 5:** Comparison of flour. (** *p* ≤ 0.05 and * *p* ≤ 0.01) indicates the elements whose means present a significant difference in a two-tailed *t*-test comparison between the organic and conventional products. Both groups in the *t*-test contained 13 samples. LOD and LOQ indicate values ≤LOQ (presence of trace) or values ≤LOD (not detectable). The values are reported in mg/kg dry weight.

		Organic Flours		Conventional Flours	
Element	*p*-Value (t)	Mean ± SD	Min–Max	Mean ± SD	Min–Max
**Ag**	0.297 (1.067)	LOQ ± 0.002	LOD–0.006	LOQ ± 0.003	LOD–0.007
Al (*)	0.023 (2.431)	11.869 ± 10.517	5.225–44.802	4.233 ± 1.379	2.295–7.391
**As**	0.114 (1.639)	0.063 ± 0.020	0.036–0.095	0.103 ± 0.084	0.036–0.305
B (**)	0.000 (6.543)	0.400 ± 0.467	LOD–1.106	1.464 ± 0.230	1.192–2.030
**Ba**	0.124 (1.592)	0.530 ± 0.142	0.285–0.784	0.446 ± 0.126	0.266–0.641
**Be**	0.136 (1.544)	0.002 ± 0.001	0.001–0.006	0.001 ± 0.000	0.001–0.001
**Ca**	0.242 (1.200)	447.069 ± 28.188	391.647–486.933	434.249 ± 26.244	372.960–480.070
**Cd**	0.056 (2.006)	0.017 ± 0.006	0.010–0.030	0.013 ± 0.004	0.008–0.020
**Co**	0.908 (0.117)	LOQ ± 0.003	LOD–0.010	LOD ± 0.001	LOD–0.004
**Cr**	0.054 (2.029)	0.117 ± 0.092	0.046–0.382	0.061 ± 0.029	0.037–0.149
**Cu**	0.245 (1.193)	2.724 ± 0.509	2.000–3.861	2.924 ± 0.326	2.401–3.426
Fe (*)	0.031 (2.289)	19.134 ± 8.469	11.067–45.423	13.320 ± 2.018	10.950–17.896
K (*)	0.038 (2.197)	1910.892 ± 172.820	1664.408–2246.599	2051.040 ± 151.758	1832.890–2303.912
**Mg**	0.388 (0.878)	390.328 ± 30.020	329.942–424.400	380.778 ± 25.209	343.350–426.213
**Mn**	0.879 (0.154)	9.434 ± 1.645	6.734–11.814	9.343 ± 1.349	7.660–12.084
**Mo**	0.429 (0.804)	1.105 ± 1.003	0.551–4.341	0.875 ± 0.144	0.666–1.180
**Na**	0.173 (1.405)	28.049 ± 14.513	17.849–74.832	44.567 ± 39.018	17.774–141.929
**Ni**	0.523 (0.648)	0.161 ± 0.234	0.039–0.920	0.117 ± 0.047	0.062–0.229
**P**	0.513 (0.665)	1746.273 ± 213.606	1335.880–2115.440	1799.051 ± 190.710	1517.020–2159.440
**Pb**	0.250 (1.180)	0.088 ± 0.025	0.062–0.148	0.074 ± 0.037	0.036–0.186
**Sb**	0.765 (0.303)	LOD ± 0.001	LOD–LOD	LOD ± 0.001	LOD–LOD
Se (**)	0.000 (5.020)	0.186 ± 0.023	0.158–0.230	0.127 ± 0.036	0.091–0.200
Si (*)	0.019 (2.525)	28.860 ± 20.680	15.683–85.435	13.105 ± 4.473	10.116–27.071
**Sn**	0.285 (1.094)	0.011 ± 0.003	0.005–0.016	0.015 ± 0.011	0.006–0.035
**Ti**	0.060 (1.975)	0.104 ± 0.122	0.027–0.484	0.034 ± 0.018	0.018–0.080
**Tl**	0.342 (0.970)	0.002 ± 0.005	LOD–0.019	LOD ± 0.000	LOD–LOQ
V (*)	0.017 (2.560)	0.021 ± 0.018	0.003–0.075	0.082 ± 0.079	0.007–0.235
**Zn**	0.600 (0.531)	12.892 ± 1.982	9.403–16.102	13.264 ± 1.569	11.396–15.950

**Table 6:** Comparison of PLS-DA discriminant models across different products involved evaluating at least two models for each set:one including the log transform of the element concentrations and the other using untransformed data. Precision and accuracy [40] were evaluated by the classification toolbox class_gui. These matrices were consistently reported for both classification and cross-validated prediction. Precision of the two classes, conventional and organic, are provided.

Product	Data Pre-Treatment	Selected Elements	Number of Latent Variables (Computed; Optimal)	R^2^	Q^2^	Precision (Conv; Org)	Accuracy	Pred. Precision (Conv; Org)	Pred. Accuracy
**All samples together**	Log_10_ transform, autoscaled	B, Cd, Cu, Fe, Se	4; 4	0.78	0.76	0.98; 0.97	0.97	0.96; 0.95	0.96
**All samples together**	autoscaled	B, Cd, Cu, Se, Si	3; 2	0.74	0.72	0.93; 0.93	0.93	0.93; 0.92	0.92
**Seed**	Log_10_ transform, autoscaled	B, Cd, Cu, Fe, Mg, Se	2; 1	0.62	0.59	1;0.95	0.97	0.88; 0.89	0.89
**Seed**	autoscaled	B, Cd, Cu, Fe, Mg, Se	6; 1	0.63	0.59	1; 0.95	0.97	0.88; 0.85	0.86
**Bran**	Log_10_ transform, autoscaled	B, Cd, Co, Cu, Fe, K, Na, Pb, Se, Sn	3; 2	0.89	0.83	1; 1	1	1; 1	1
**Bran**	autoscaled	B, Cd, Co, Cu, Fe, K, Mo, Na, P, Pb, Se, Si, Sn	2; 2	0.91	0.86	1; 1	1	1; 1	1
**Semolina**	Log_10_ transform, autoscaled	Ag, B, Cd, Cu, K, Se, Si	2; 1	0.91	0.89	1; 1	1	1; 1	1
**Semolina**	autoscaled	Ag, B, Cd, Cu, K, Se	2; 1	0.89	0.87	1; 1	1	1; 1	1
**Flour**	Log_10_ transform, autoscaled	B, K, Se	3; 1	0.8	0.73	0.92; 0.92	0.92	0.85; 0.95	0.85
**Flour**	Log10 transform, autoscaled	Al, B, Cd, Cr, Fe, K, Se, Si, V	3; 2	0.84	0.71	1; 1	1	1; 0.93	0.96
**Flour**	autoscaled	B, K, Se	3; 1	0.81	0.75	0.92; 0.92	0.92	0.92; 0.92	0.92
**Flour**	autoscaled	Al, B, Cd, Co, Cr, Cu, Fe, K, Na, Se, Si, V	12; 1	0.79	0.75	1; 1	1	0.93; 1	0.96

## Data Availability

The original contributions presented in the study are included in the article/Supplementary Materials, further inquiries can be directed to the corresponding author.

## Supplementary Materials

The following supporting information can be downloaded at: https://www.mdpi.com/article/10.3390/foods13121924/s1, Table S1: Elements determined and wavelengths used for their quantification. The parameters are computed on all the product samples as a whole: seed, bran, semolina and flour in a unique set. (**) indicates the elements, whose means, give a significant two tailed *t*-test comparison (α = 0.05) between organic products and the conventional ones. Figure S1: Plots of Pearson’s correlation coefficient between element concentrations within the product sets. Statistically significant (*p*-value ≤ 0.05 in a two tails *t*-test) coefficient are boxed within the plots.

## Appendix A

Soils were randomly sampled throughout the year, resulting in 40 samples: 16 from conventionally cultivated parcels and 24 from organically cultivated parcels. Their pH is approximately 8.0. Table A1 shows the values of the elements measured in these soil samples. The mineralization and quantification procedure adopted was identical to the one previously described.

It is remarkable that the soil has very low content, less than LOD, of Ag, Sb, and Se, and only minimal traces of TI. The relatively high values of Al, Cr, Fe, and Cu may be due to some pollution from nearby railway tracks and mechanical industrial facilities. The basic pH can prevent the high amount of Al and Mn from carrying out their toxic effect on wheat plants [42,43]. Comparison, through monovariate *t*-tests, of organic and conventional soils shows a meaningful difference, at the 0.05 significance level, for the following elements: Ba, Be, Cd, Cr, Cu, Fe, Mn, Ni, V, and Zn.

**Table A1:** Soil elemental content. (**) indicates the elements that give a significant *t*-test comparison (α = 0.05) between the average values of soil samples for organic cultivation and those for conventional cultivation. LOD and LOQ are to indicate a value ≤LOQ (presence of trace) or a value ≤LOD (not detectable). The scale of the values is mg/kg dry weight.

			All Soil Samples		Soils from Organic Cultivation		Soils from Conventional Cultivation	
	LOD	LOQ	Min–Max	Mean ± SD	Min–Max	Mean +SD		Mean +SD
**Ag**	4 × 10^−4^	0.001	LOD–LOD	LOD ± 0.000	LOD–LOQ	LOD ± 0.000	LOD–LOQ	LOD ± 0.000
**Al (**)**	0.001	0.003	1488.550–4140.450	2092.514 ± 670.361	1488.550–4140.450	2167.355 ± 451.637	1560.910–3189.510	1980.254 ± 466.345
**As**	0.001	0.004	1.000–3.343	2.322 ± 0.612	1.366–3.073	2.219 ± 0.679	1.000–3.343	2.476 ± 0.701
**B**	0.830	2.767	66.027–144.156	91.205 ± 18.454	66.027–125.625	86.689 ± 19.376	74.560–144.156	97.978 ± 18.515
**Ba (**)**	8 × 10^−5^	3×10^−4^	140.287–233.653	189.969 ± 21.746	140.287–228.053	183.784 ± 17.618	168.206–233.653	199.247 ± 17.175
**Be (**)**	3 × 10^−5^	1×10^−4^	1.249–2.093	1.690 ± 0.236	1.249–2.003	1.563 ± 0.167	1.585–2.093	1.879 ± 0.144
**Ca**	0.009	0.029	2231.142–2993.378	2436.184 ± 176.779	2231.142–2993.378	2441.475 ± 162.532	2261.407–2840.278	2428.246 ± 167.641
**Cd (**)**	2 × 10^−4^	6×10^−4^	0.132–0.304	0.223 ± 0.041	0.167–0.304	0.235 ± 0.048	0.132–0.291	0.205 ± 0.047
**Cr (**)**	4 × 10^−4^	0.001	53.245–96.209	76.454 ± 11.511	53.245–93.176	70.706 ± 7.699	70.934–96.209	85.076 ± 6.785
**Cu (**)**	4 × 10^−4^	0.001	26.933–48.164	35.780 ± 4.645	26.933–41.021	33.726 ± 4.346	31.228–48.164	38.860 ± 4.470
**Fe (**)**	2 × 10^−4^	6×10^−4^	13,698.050–21,144.190	17,352.365 ± 2128.555	13,698.050–20,820.220	16,409.707 ± 1624.874	16,299.890–21,144.190	18,766.351 ± 1596.296
**K**	2 × 10^−4^	8×10^−4^	755.832–1222.931	963.475 ± 85.755	755.832–1118.040	947.159 ± 102.945	774.873–1222.931	987.948 ± 105.488
**Mg**	0.001	0.005	1990.411–2677.165	2269.414 ± 175.033	1990.411–2612.228	2240.159 ± 165.078	2148.065–2677.165	2313.296 ± 170.404
**Mn (**)**	5 × 10^−5^	2×10^−4^	693.361–1277.959	931.030 ± 147.022	693.361–1277.959	885.505 ± 91.233	883.112–1147.556	999.318 ± 84.972
**Mo**	6×10^−4^	0.002	0.660–5.678	1.262 ± 1.012	0.660–5.678	1.212 ± 0.944	0.806–4.693	1.337 ± 0.968
**Na**	0.001	0.004	334.815–802.104	550.969 ± 95.093	456.723–712.040	543.160 ± 118.666	334.815–802.104	562.683 ± 121.713
**Ni (**)**	5 × 10^−4^	0.002	39.274–76.972	53.301 ± 10.004	39.274–76.972	49.046 ± 7.288	48.823–74.643	59.683 ± 6.668
**P**	0.004	0.012	98.932–871.575	680.919 ± 159.183	103.282–871.575	715.453 ± 163.216	98.932–786.637	629.120 ± 167.303
**Pb**	7 × 10^−4^	0.002	13.623–43.143	19.372 ± 4.417	13.623–43.143	19.043 ± 1.897	16.091–23.817	19.866 ± 1.959
**Sb**	0.003	0.010	LOD–LOQ	LOD ± 0.001	LOD–LOQ	LOD ± 0.001	LOD–LOD	LOD ± 0.001
**Se**	0.003	0.010	LOD–LOQ	LOD ± 0.001	LOD–LOQ	LOD ± 0.001	LOD–LOQ	LOD ± 0.001
**Si**	0.018	0.060	197.230–2826.853	963.128 ± 830.102	218.109–2588.145	927.417 ± 889.582	197.230–2826.853	1016.695 ± 902.656
**Sn**	0.007	0.023	0.962–655.190	34.511 ± 136.265	1.161–655.190	30.777 ± 140.891	0.962–584.401	40.113 ± 145.189
**Tl**	0.001	0.003	LOD–1.239	0.494 ± 0.338	LOD–1.239	0.469 ± 0.298	LOD–0.890	0.530 ± 0.307
**V (**)**	2 × 10^−4^	5×10^−4^	42.138–76.322	58.657 ± 6.575	42.138–63.256	55.289 ± 5.513	55.762–76.322	63.710 ± 5.165
**Zn (**)**	7 × 10^−5^	2×10^−4^	64.296–96.585	80.211 ± 9.031	64.296–94.897	75.987 ± 6.488	75.810–96.585	86.545 ± 6.163
