# Supplementary material for: Distribution of Elements in Durum Wheat Seed and Milling Products: Discrimination between Cultivation Methods through Multivariate Data Analysis
Source: Foods. 2024 Jun 18;13(12):1924. doi: 10.3390/foods13121924 (PMC11203146; doi:10.3390/foods13121924)
Supplement: Supplementary file 1 [file foods-13-01924-s001.zip › foods-3032751-supplementary.pdf]

Supplementary material

Table S1: Elements determined and wavelengths used for their quantification. The parameters are computed on all the product samples as a whole: seed, bran, semolina and flour in a unique set. (**) indicates the elements, whose means, give a significant two tailed t-test comparison (α=0.05) between organic products and the conventional ones.

				Organic			Conventional		
**Element**	**Wavelength (nm)**	**LOD (mg/Kg)**	**LOQ (mg/Kg)**	**Mean±SD (mg/Kg)**	**Median (mg/Kg)**	**Min – Max (mg/Kg)**	**Mean±SD (mg/Kg)**	**Median (mg/Kg)**	**Min – Max (mg/Kg)**
**Ag (**)**	328.068	7×10^{-4}	0.002	0.003 ± 0.002	LOQ	LOD - 0.007	0.004 ± 0.003	0.003	LOD - 0.013
**Al (**)**	396.152	3×10^{-4}	0.001	6.940 ± 5.875	5.311	1.788 - 44.802	4.089 ± 1.517	4.117	1.447 - 7.705
**As**	189.042	0.002	0.007	0.069 ± 0.029	0.067	0.021 - 0.182	0.086 ± 0.066	0.065	0.020 - 0.363
**B (**)**	249.773	8×10^{-4}	0.003	1.182 ± 0.890	1.250	LOD - 2.502	1.894 ± 0.624	1.844	1.109 - 3.185
**Ba**	493.409	3×10^{-5}	1×10^{-4}	0.748 ± 0.367	0.626	0.285 - 1.953	0.688 ± 0.403	0.584	0.266 - 2.057
**Be**	313.042	2×10^{-5}	6×10^{-5}	0.001 ± 0.001	0.001	LOD - 0.006	0.001 ± 0.001	0.001	LOD - 0.003
**Ca**	393.366	0.007	0.024	460.897 ± 31.085	460.871	391.647 - 519.332	456.418 ± 38.296	457.874	372.960 - 531.502
**Cd (**)**	214.438	1×10^{-4}	4×10^{-4}	0.024 ± 0.011	0.021	0.010 - 0.053	0.018 ± 0.008	0.017	0.008 - 0.036
**Co**	228.616	0.001	0.003	LOQ ± 0.003	LOQ	LOD - 0.011	LOQ ± 0.003	LOQ	LOD - 0.012
**Cr**	267.716	7×10^{-4}	0.002	0.090 ± 0.054	0.083	0.034 - 0.382	0.081 ± 0.077	0.072	0.024 - 0.615
**Cu (**)**	327.396	8×10^{-4}	0.003	3.579 ± 1.131	3.535	2.000 - 5.848	4.065 ± 1.481	3.964	2.138 - 7.457
**Fe**	259.940	4×10^{-4}	0.001	24.698 ± 10.022	25.202	9.983 - 48.132	21.413 ± 10.410	22.031	7.844 - 41.784
**K**	769.896	0.003	0.011	2679.148 ± 882.771	2968.295	1377.052 - 4242.568	2833.772 ± 969.482	3072.834	1623.165 - 4540.521
**Mg**	279.553	1×10^{-4}	4×10^{-4}	463.610 ± 91.025	504.347	300.566 - 603.480	453.129 ± 96.758	488.754	314.100 - 603.324
**Mn**	259.373	1×10^{-4}	3×10^{-4}	26.278 ± 16.906	29.341	6.734 - 61.671	26.906 ± 19.292	28.613	7.456 - 73.585
**Mo**	202.030	7×10^{-4}	0.002	0.953 ± 0.502	0.852	0.539 - 4.341	0.994 ± 0.260	0.935	0.659 - 1.644
**Na (**)**	588.995	0.001	0.004	28.037 ± 10.448	25.428	16.225 - 74.832	36.871 ± 25.143	25.317	15.214 - 141.929
**Ni**	231.604	0.001	0.004	0.161 ± 0.138	0.139	0.027 - 0.920	0.161 ± 0.098	0.143	0.041 - 0.580

**P**	177.495	0.001	0.004	2725.118 ± 1078.984	3082.185	1286.050 - 4748.610	2803.837 ± 1200.185	3065.615	1337.920 - 4985.480
**Pb**	220.353	0.004	0.012	0.076 ± 0.027	0.072	0.036 - 0.148	0.070 ± 0.036	0.066	0.026 - 0.263
**Sb**	217.581	0.005	0.015	LOD ± 0.004	LOD	LOD - 0.019	LOD ± 0.003	LOD	LOD - 0.020
**Se (**)**	196.090	0.001	0.005	0.217 ± 0.038	0.217	0.151 - 0.290	0.148 ± 0.039	0.155	0.077 - 0.222
**Si**	251.611	8×10^{-5}	3×10^{-4}	23.961 ± 14.109	21.451	6.314 - 85.435	19.116 ± 13.210	15.465	4.997 - 69.089
**Sn**	189.989	3×10^{-4}	9×10^{-4}	0.007 ± 0.003	0.006	0.003 - 0.016	0.008 ± 0.010	0.005	0.002 - 0.059
**Ti (**)**	323.452	4×10^{-4}	0.001	0.051 ± 0.068	0.032	0.007 - 0.484	0.031 ± 0.019	0.025	0.007 - 0.099
**Tl**	190.856	5×10^{-4}	0.002	0.003 ± 0.007	LOD	LOD - 0.033	0.004 ± 0.009	LOD	LOD - 0.043
**V (**)**	292.402	6×10^{-4}	0.002	0.012 ± 0.011	0.010	LOQ - 0.075	0.035 ± 0.080	0.010	LOQ - 0.520
**Zn**	202.548	6×10^{-4}	0.002	23.564 ± 11.829	25.262	8.952 - 45.948	23.629 ± 12.273	24.680	9.035 - 47.971

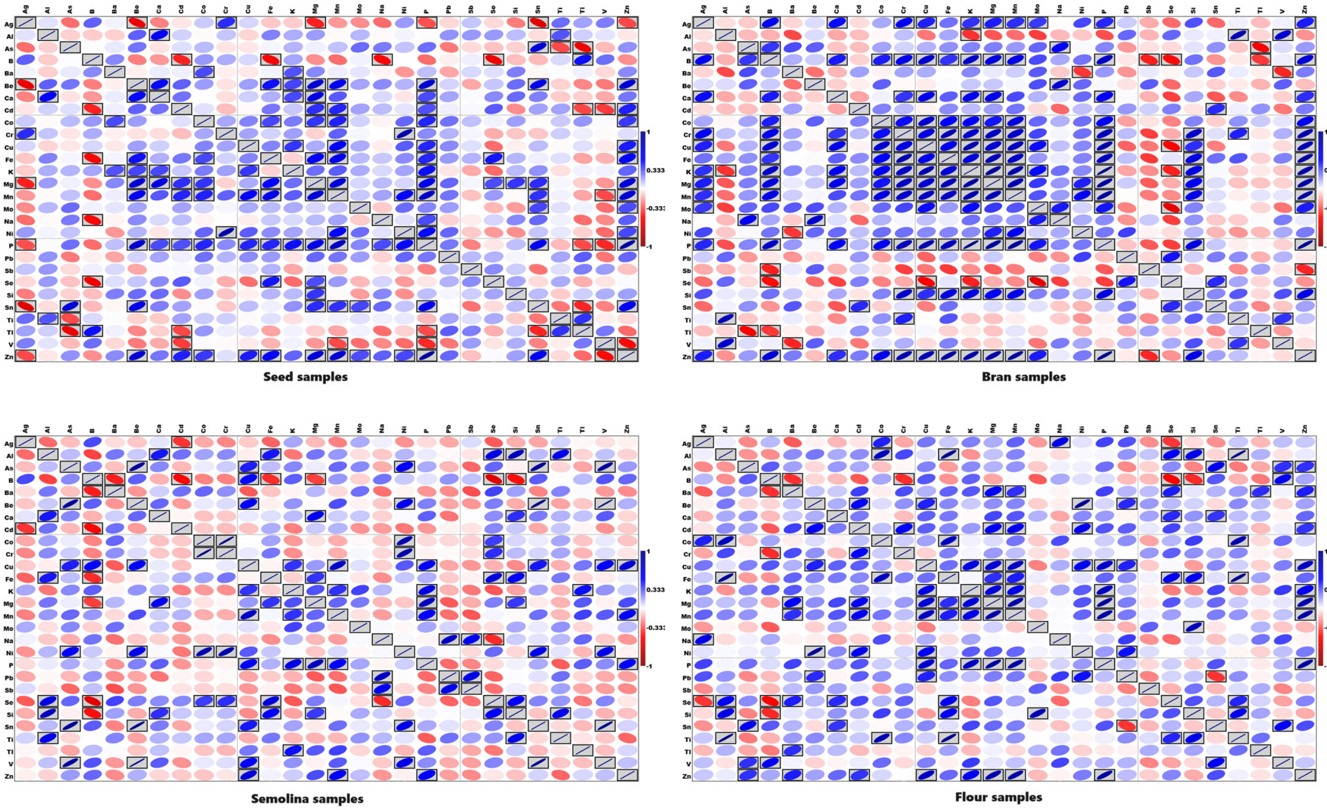

Figure S1. Plots of Pearson's correlation coefficient between element concentrations within the product sets. Statistically significant (p-value≤0.05 in a two tails t-test) coefficient are boxed within the plots.
